# The role of electronic medical records in improving health care quality: A quasi-experimental study

**DOI:** 10.1097/MD.0000000000029627

**Published:** 2022-07-29

**Authors:** Ariff Azfarahim Ibrahim, Mohd ‘Ammar Ihsan Ahmad Zamzuri, Rosnah Ismail, Ahmad Husni Ariffin, Aniza Ismail, Muhamad Hazizi Muhamad Hasani, Mohd Rizal Abdul Manaf

**Affiliations:** a Department of Community Health, Faculty of Medicine, Universiti Kebangsaan Malaysia, Fakulti Perubatan UKM, Cheras, Kuala Lumpur, Malaysia; b Family Health Development Division, Seremban District Health Office, Ministry of Health, Seremban, Negeri Sembilan, Malaysia.

**Keywords:** electronic medical records, patient satisfaction, PSQ-18

## Abstract

The Teleprimary Care—Oral Health Clinical Information System (TPC-OHCIS) is an updated electronic medical record (EMR) that has been applied in Malaysian primary healthcare. Recognizing the level of patient satisfaction following EMR implementation is crucial for assessing the performance of health care services. Hence, the main objective of this study was to compare the level of patient satisfaction between EMR-based clinics and paper-based clinics.

The study was a quasi-experimental design that used a control group and was conducted among patients in 14 public primary healthcare facilities in the Seremban district of Malaysia from May 10, to June 30, 2021. Patient satisfaction was assessed using the validated Short-Form Patient Satisfaction Questionnaire, which consisted of 7 subscales. All data were analyzed using the IBM Statistical Package for Social Sciences version 21.

A total of 321 patients consented to participate in this study, and 48.9% of them were from EMR clinics. The mean score for the communication subscale was the highest at 4.08 and 3.96 at EMR-adopted clinics and paper-based record clinics. There were significant differences in general satisfaction and communication subscales, with higher patient satisfaction found in clinics using EMR.

With the utilization of EMR, patient satisfaction and communication in delivering healthcare services have improved.

## 1. Introduction

Electronic medical records (EMRs) are digitalized systems for maintaining patient records that have become extensively employed worldwide. In 2009, the United States passed the Health Information Technology for Economic and Clinical Health Act, which sets a precedent in health information technology by providing incentive payments to healthcare providers who adopted the EMR.^[1]^ Healthcare stakeholders believe that the growing use of EMR will eventually enhance the quality of medical care by reducing medical mistakes, minimizing duplication errors, reducing unnecessary diagnostic procedures, and making data collection and accessibility easier, thus increasing overall satisfaction.^[2]^ Nevertheless, research conducted in Saudi Arabia has indicated that using EMR has successfully enriched numerous areas of the healthcare system, including physician productivity, information availability, and healthcare service quality.^[3]^

According to the Institute of Medicine, the widespread use of the EMR is very critical to improve healthcare quality. The World Health Organization defines the quality of care as the extent to which health care services that are provided to individuals and patient populations can improve the desired health outcomes.^[4]^ Additionally, healthcare quality is a critical component in delivering services within a healthcare institution. An eminent physician named Avedis Donabedian, who proposed a standard framework for the measurement of healthcare quality in 1966, was the pioneer of healthcare quality.^[5]^ The patient satisfaction domain, according to Donabedian’s quality assessment model, is defined as a patient-reported outcome measurement. This is as opposed to patient-reported experiences, which are more related to the structures and procedures of treatment.

In the era of technological advancement, adopting an EMR to be integrated into primary care services using the most up-to-date information and communication technologies system is inevitable and deemed necessary. Similarly, it is critical to measure the quality of patient care and health outcomes by assessing the level of patient satisfaction following EMR implementation. This is imperative because patient satisfaction evaluation will allow for a thorough and fair judgment of the structure and technique as well as the outcome of health services from the patients’ perspective.

As an upper-middle-income country, Malaysia does not lag behind in the endeavor of healthcare digitalization transformation, as it was among the first Asian countries to implement EMR through the establishment of Telemedicine Blueprint in 1997.^[6]^ Subsequently, in 2017, an updated EMR called the Teleprimary Care – Oral Health Clinical Information System (TPC-OHCIS) was implemented by the government to serve public primary healthcare services. The TPC-OHCIS is an integrated system between health clinics and dentistry clinics with the ultimate purpose of boosting the efficiency of healthcare delivery and ensuring the continuity of patient care based on the “womb to tomb” model.^[7]^ Despite the fact that it has been several years of implementation in Malaysia, evidence of service improvement following EMR utilization remains scarce. To the best of our knowledge, this is the first study to venture into an EMR postimplementation. Nevertheless, recognizing the level of patient satisfaction following EMR implementation is crucial in measuring the effectiveness of patient care and health outcomes. Hence, the main objective of this study was to compare the level of patient satisfaction between clinics that adopted EMR and conventional clinics that used paper-based records. The outcome of this study can be used by policymakers to rectify any shortfalls of EMR implementation and perhaps use the information to expand the coverage of EMR usage. The authors believe patient satisfaction in EMR clinics is significantly higher than in clinics without EMR. To test our hypothesis, we have conducted a quasi-experimental study in a setting where the utilization of EMR is robust, thus presented below in a comprehensive structure.

## 2. Method

### 2.1. Study setting

The study was conducted in Negeri Sembilan, as it was the first state to receive the honor to implement an updated EMR in 2017. The state is situated in the central region of Malaysia, on the outskirt of the Federal Territory of Kuala Lumpur and Selangor (the most densely populated region). Seremban district was chosen as the study location because it is the only district that has EMR services. There are 14 public primary healthcare providers within the Seremban district, of which 6 primary healthcare providers are equipped with an EMR system, while the remaining 8 primary healthcare providers maintain the conventional paper-based record. These 14 healthcare facilities are under the jurisdiction of a single authority named the Seremban District Health Office. The office oversees the allocation of both types of facilities in terms of human and financial resources, thus ensuring distribution equality.

### 2.2. Study design

The study was a quasi-experimental posttest design that used a control group. The quasi-experimental methodology was chosen because it is part of the researchers’ main study frame. Nevertheless, a quasi-experimental design was performed, as it is commonly used to assess medical informatics intervention.^[8]^ The design was utilized to measure the impact of EMR adoption on patients’ perceptions of the quality of healthcare services. All 14 public primary healthcare facilities were included in the study to compare facilities that adopted EMR with those that did not. The intervention group in this study consisted of participants who rated the Short-Form Patient Satisfaction Questionnaire (PSQ-18) questionnaire for public primary healthcare with EMR, whereas the control group consisted of participants who rated the PSQ-18 questionnaire for primary healthcare without EMR.

### 2.3. Sample selection

A nonprobability convenience sampling method was used to select participants. OpenEpi Calculator was used to calculate the sample size of the study by comparing 2 means.^[9]^ The error α was set to 5%. The power is set at 80. The required sample was 362, after adjusting for an attrition rate of 20%. The inclusion criteria for this study were patients aged 15-year-old and above, nonilliterate, and Malaysian citizens. On the other hand, the exclusion criteria were patients who did not give consent, those who attended the emergency room, those who came with severe pain as the chief presenting complaint, and those who did not understand either Malay language or English language.

### 2.4. Data collection

A self-administered questionnaire consisting of 2 parts was administered from May 10, 2021, to June 30, 2021. The first part included items on sociodemographic factors (gender, age, marital status, household income, employment status, education level, type of residence, and purpose of visiting the clinic). To explore patient satisfaction with healthcare services, we used the validated PSQ-18 originally developed by Marshall and Hays.^[10]^ PSQ-18 comprises 18 items with 7 dimensions that measure general satisfaction (2 items), technical quality (4 items), interpersonal manner (2 items), communication (2 items), financial aspects (2 items), time spent with doctors (2 items), and accessibility and convenience (4 items). These items are scored on a 5-point Likert scale ranging from 1 (strongly agree) to 5 (strongly disagree). Some PSQ-18 items were worded so that agreement reflects satisfaction with medical care, whereas other items were worded so that agreement reflects dissatisfaction with medical care. We reversed the scores of the items that reflected disagreement with medical care to tabulate the total satisfaction score. Higher scores reflect greater satisfaction with medical care. After item scoring, the items within the same subscale were averaged to create 7 subscale scores.

### 2.5. Data analysis

Data were tabulated in Microsoft Excel, and analysis was performed using the Statistical Package for Social Sciences (SPSS) 21.0. To describe the participants’ demographic data, descriptive statistics such as means, frequencies, and standard deviations were calculated. The Kolmogorov–Smirnov test was used to test the normality of the data prior to bivariate analysis. The nonparametric Mann–Whitney *U* test was used in view of the violation of the normal distribution to compare both intervention and control groups.

### 2.6. Ethical considerations

The study was approved by the Medical Research & Ethics Committee of the Ministry of Health Malaysia (KKM/NIHSEC/P20-1047) and registered with the National Medical Research Register. The questionnaire was anonymous, and verbal informed consent was obtained from all participating patients prior to enrollment in the study. This study was conducted in accordance with the principles of the Declaration of Helsinki.

## 3. Results

A total of 321 patients (88.7% response rate) consented to participate in this study, with 157 (48.9%) from the EMR-adopted clinic. Table 1 presents a descriptive analysis of the respondents. Of total number of respondents, 98 (30.5%) were male, and 223 (69.5%) were female. The mean and standard deviation age of the patients was 37.4 (±11.9) years. The majority of the patients were married, 241 (75.1%) were married, and 245 (76.3%) were Malay. More than half of the total patients had tertiary education 166 (51.7%), and 208 (64.8%) were employed with a monthly household income of Malaysian Ringgit 2000 or less 121 (37.7%). Most of the patients resided in an urban area (243; 75.7%) and came to the clinic for follow-up treatment (184; 57.3%).

**Table 1:** Descriptive analysis of study population.

**Characteristics**	**EMR-adopted clinics (%**) **N = 157**	**Paper-based record clinics (%**) **N = 164**	**Total (%**) **(N = 321**)
Age,* yr, mean (± SD)	35.2 (± 11.0)	39.5 (± 12.3)	37.4 (± 11.9)
Gender			
Male	47 (29.9)	51 (31.1)	98 (30.5)
Female	110 (70.1)	113 (68.9)	223 (69.5)
Marital status			
Married	106 (67.5)	135 (82.3)	241 (75.1)
Single	51 (32.5)	29 (17.7)	80 (24.9)
Highest education level			
Secondary level	73 (46.5)	82 (50.0)	155 (48.3)
Tertiary level	84 (53.5)	82 (50.0)	166 (51.7)
Race			
Malay	111 (70.7)	134 (81.7)	245 (76.3)
Non-Malay	46 (29.3)	30 (18.3)	76 (23.7)
Occupation			
Employed	111 (70.7)	97 (59.2)	208 (64.8)
Unemployed	39 (24.8)	54 (32.9)	93 (29.0)
Pensioner	7 (4.5)	13 (7.9)	20 (6.2)
Residence			
Urban	133 (84.7)	110 (67.1)	243 (75.7)
Rural	24 (15.3)	54 (32.9)	78 (24.3)
Purpose of visit			
Follow-up	79 (50.3)	105 (64.0)	184 (57.3)
New case	28 (17.8)	32 (19.5)	60 (18.7)
Others	50 (31.9)	27 (16.5)	77 (24.0)
Monthly household income (MYR)			
<2000	61 (38.9)	60 (36.6)	121 (37.7)
2001–4000	60 (38.2)	45 (27.4)	105 (32.7)
>4001	36 (22.9)	59 (36.0)	95 (29.6)

The mean satisfaction rating for each subscale in the 2 types of clinics is shown in Table 2. Our study found that high satisfaction was achieved in all subscales except for the time spent with the doctor’s subscale, where mean scores for this subscale were lowest at 3.55 (SD 0.64) and 3.54 (SD 0.61) at EMR-adopted clinics and paper-based record clinics respectively. The mean score for communication was the highest at 4.08 (SD 0.59) and 3.96 (SD 0.60) at EMR-adopted clinics and paper-based record clinics, respectively.

**Table 2:** Mean score of 7 subscale patients’ satisfaction towards clinic adopted EMR compared to the paper-based record clinics of patients attending fourteen public primary healthcare clinics in Seremban District of Malaysia.

**Subscale**	**EMR adopted clinics,** **mean (SD**)	**Paper-based record clinics,** **mean (SD**)
General satisfaction	4.08 (0.59)	3.81 (0.63)
Technical quality	3.76 (0.57)	3.64 (0.54)
Interpersonal manner	3.70 (0.68)	3.59 (0.62)
Communication	4.08 (0.59)	3.96 (0.60)
Financial aspects	3.92 (0.68)	3.79 (0.68)
Time spent with doctor	3.55 (0.64)	3.54 (0.61)
Accessibility and convenience	3.68 (0.60)	3.66 (0.55)

To answer the objective of this study, the Mann–Whitney *U* test was used to calculate the satisfaction of the 7 subscales, as presented in Table 3. The findings of this study demonstrated that only 2 out of 7 subscales of patient satisfaction had significant differences in patient satisfaction due to the implementation of EMR. The results indicated that general satisfaction and communication in EMRs adopted clinics were substantially higher compared to paper-based record clinics with a *P* value of <.05.

**Table 3:** Mann–Whitney *U* test results for 7 subscale patients’ satisfaction towards clinic adopted EMR compared to the paper-based record clinics of patients attending fourteen public primary healthcare clinics in Seremban District of Malaysia.

**Subscale**	**Type of clinic**	**N**	**Mean rank**	**Sum of**	**U**	***P* value**
General satisfaction	With EMR Without EMR	157 164	171.9 150.6	26,986.5 24,694.5	11,164.5	.03
Technical quality	With EMR Without EMR	157 164	168.1 154.2	26,389.0 25,292.0	11,762.0	.17
Interpersonal manner	With EMR Without EMR	157 164	168.5 153.8	26,461.5 25,219.5	11,689.5	.14
Communication	With EMR Without EMR	157 164	171.0 151.4	26,847.5 24,833.5	11,303.5	.04
Financial aspects	With EMR Without EMR	157 164	170.2 152.2	26,716.5 24,964.5	11,434.5	.07
Time spent with doctor	With EMR Without EMR	157 164	159.5 162.4	25,041.0 26,640.0	12,638.0	.76
Accessibility and convenience	With EMR Without EMR	157 164	161.9 160.2	25,417.0 26,264.0	12,734.0	.86

## 4. Discussion

The TPC-OHCIS is one of the latest EMR systems applied in public primary healthcare by the Ministry of Health, Malaysia. Initially, the EMR system in Malaysia began in the early 1990s at the tertiary center. However, the expansion of the system into public primary healthcare was slow, and the progress took years to reach. Although the services came into effect in 2005, integration with the dental clinic system only materialized in the mid of 2017. As stated, the implementation of the TPC-OHCIS was pilot tested for the first time at 6 public primary healthcare facilities in the Seremban district of Malaysia beginning in mid of the year 2017.^[6]^

In the current study, the researchers aimed to compare patient satisfaction between clinics adopting EMR and paper-based record clinics among patients attending 14 public primary healthcare facilities in the Seremban district of Malaysia. The quasi-experimental posttest study was utilized because our study was about medical informatics. This research design is frequently used to evaluate public health interventions that do not require randomization.^[8]^ Because of the difficulties in randomizing individuals, locations, and pretest data too late, this approach was chosen.^[11]^ However, it can also provide several advantages for the study, such as fewer resources are needed, including cost-effectiveness, and the ability to evaluate the effectiveness of EMR in the real world carried out by health personnel.^[12]^

The findings in this study demonstrated that only 2 out of 7 subscales of patient satisfaction significantly improved patient satisfaction following the implementation of EMR. The results indicated that the general satisfaction in EMRs adopted by primary healthcare was substantially higher than that in paper-based record primary healthcare. The current result is similar to the findings of a study conducted in Kuwait and Qatar, which showed that the general levels of patient satisfaction were higher with EMR implementation.^[13,14]^ In other words, the use of EMR in the study setting was able to improve health care services provided to individuals and patient populations despite knowing that public primary healthcare usually had a high number of patients who were unable to provide ample time for consultation.^[15]^

In the current study, the communication domain had a high level of satisfaction. According to reports, patients prefer a compassionate and friendly physician to a professional physician. Physicians’ communication style, which emphasizes decency, warmth, and displaying respect and care, plays a major role, and has the highest impact on patient satisfaction.^[16]^ On the other hand, unsatisfactory communication can lead to an increase in complaints, lawsuits, and disenrollment. Communication between doctors and patients is essential in primary healthcare since most patients attend the same clinic periodically for their follow-up appointments. Demonstrating a good relationship between physicians and patients aids in the collection of systematic data, formulation of an appropriate diagnosis and treatment plan, and improvement of the overall outcome.^[17]^ The results of this study strongly support that EMR might be a beneficial tool for facilitating communication, promoting a better knowledge of patients’ concerns, and providing a holistic approach to managing their health problems.^[18]^ A systematic review of the impact of EMR on doctor–patient communication concluded that there were no changes in patients or at least 5 out of 22 studies reported positive changes in patient satisfaction.^[19]^ The same result was found in a qualitative study conducted in Lebanon, which reported that EMR did not negatively affect physician–patient communication once the physicians were able to adapt to the technology.^[20]^ In fact, clinics with paper-based records are much more time-consuming, and a higher risk of misinformation may lead to interruption in communication between the physician and the patient. This scientific evidence clearly shows that patient perceptions are not affected by EMRs. In contrast, EMR has the ability to motivate healthcare providers once it is fully adopted and compiled.

### 4.1. Strength and limitation

This study represents a novel approach to public primary healthcare in Malaysia owing to the maturity of the EMR systems in the country. Second, this study revealed that patients attending public primary healthcare fully support an EMR system that can improve their health outcomes. Furthermore, these findings should prompt healthcare stakeholders to expand the system nationwide as soon as possible. However, one of the limitations of this study is that the results cannot be generalized freely because of the disadvantage of the sampling technique used, which was not probabilistic in nature. Nevertheless, due to the shortage of time, limited resources, and the fact that a sufficient number of respondents were enrolled, they inevitably reduced the power of the study. In addition, the timing of the study conducted during the upsurge trend of coronavirus disease 2019 cases might have caused the disproportionate number of attendances to the health facilities because strict standard operating procedures were applied, especially in conventional paper-based clinics that usually saw an abundance of patients.

## 5. Conclusion

This research aided in the discovery of in-depth facts about patient satisfaction in public primary healthcare services. This study will serve as the foundation for future studies by adding cost analysis of EMR, as the cost of integration to the new system has always been an issue. With the use of EMRs, patient satisfaction and communication in delivering healthcare services have improved. We believe that this constellation of findings will provide factual information on a particular aspect that needs improvement while auditing and reviewing primary healthcare in delivering its services. Lastly, these findings should encourage policymakers to implement the system nationwide in a significant effort to provide a nation with better healthcare.

## Acknowledgments

The authors would like to thank the Director-General of Health, Malaysia, for permission to publish this article.

## Author contributions

Conceptualization: Ariff Azfarahim Ibrahim and Mohd Rizal Abdul Manaf;

Methodology: Mohd ’Ammar Ihsan Ahmad Zamzuri;

Validation: Ahmad Husni Ariffin and Muhamad Hazizi Muhamad Hasani;

Formal analysis: Ariff Azfarahim Ibrahim and Mohd ’Ammar Ihsan Ahmad Zamzuri;

Writing: Ariff Azfarahim Ibrahim and Mohd ’Ammar Ihsan Ahmad Zamzuri;

Supervision: Rosnah Ismail and Aniza Ismail; Project administration: Mohd Rizal Abdul Manaf;

All authors have read and agreed to the published version of the manuscript.
